# Cause-specific mortality following sustained virologic response in hepatitis C patients treated with direct-acting antivirals: a standardized mortality ratio analysis

**DOI:** 10.1038/s41598-025-29972-6

**Published:** 2025-11-27

**Authors:** Seiichi Mawatari, Shiroh Tanoue, Kotaro Kumagai, Kohei Oda, Ai Toyodome, Haruka Sakae, Kunio Fujisaki, Yukiko Inada, Hirofumi Uto, Akiko Saisyoji, Yasunari Hiramine, Kazuaki Tabu, Sho Ijuin, Takeshi Hori, Tsutomu Tamai, Akihiro Moriuchi, Shuji Kanmura, Akio Ido

**Affiliations:** 1 Digestive and Lifestyle Diseases, Department of Human and Environmental Sciences, Kagoshima University Graduate School of Medical and Dental Sciences, 8-35-1 Sakuragaoka, Kagoshima, 890-8544 Japan; 2 Department of Epidemiology and Preventive Medicine, Kagoshima University Graduate School of Medical and Dental Sciences, 8-35-1 Sakuragaoka, Kagoshima, 890-8544 Japan; 3 Department of Hepatology, Kirishima Medical Center, 3320 Hayato-cho, Kirishima, Kagoshima 899-5112 Japan; 4 Center for Digestive and Liver Diseases, Miyazaki Medical Center Hospital, 2-16 Takamatsu-cho, Miyazaki, 880-0003 Japan; 5 Department of Hepatology, Kagoshima Kouseiren Hospital, 1-13-1 Yojirou, Kagoshima, 890-0062 Japan; 6 Department of Gastroenterology and Hepatology, Kagoshima City Hospital, 37-1 Uearata-cho, Kagoshima, 890-8760 Japan; 7 Department of Gastroenterology, National Hospital Organization Kagoshima Medical Center, 8-1 Shiroyama-cho, Kagoshima, 892-0853 Japan; 8 Kagoshima University, 1-21-24 Korimoto, Kagoshima, 890-8580 Japan

**Keywords:** HCV, DAAs, SVR, SMR, Mortality, Cardiovascular disease, Cancer, Diseases, Gastroenterology, Oncology

## Abstract

Direct-acting antivirals (DAAs) achieve high sustained virologic response (SVR) rates in patients with hepatitis C virus (HCV) infection. However, the long-term prognosis and cause-specific mortality post-SVR remain unclear. This study analyzed 1753 HCV-infected patients without a history of hepatocellular carcinoma treatment who achieved SVR with DAAs and did not develop cancer. Liver disease-related (LDR) and non-LDR mortalities were assessed using age- and sex-adjusted standardized mortality ratios (SMRs) compared with the general population. During a mean follow-up of 59.2 months, 122 patients died. The SMR for all-cause mortality was 1.050 (*p* = 0.588). The SMRs for LDR and non-LDR mortalities were 7.819 (*p* < 0.001) and 0.566 (*p* < 0.001), respectively. The SMR for extrahepatic malignancy-related mortality was significantly lower at 0.593 (*p* = 0.013), whereas cardiovascular disease (CVD)-related mortality did not differ significantly at 0.884 (*p* = 0.554). These findings were consistent regardless of age, sex, liver cirrhosis presence, diabetes, or chronic kidney disease. Overall mortality after SVR in patients with HCV was comparable with that of the general population. Nevertheless, LDR mortality remained significantly elevated, whereas non-LDR and extrahepatic malignancy-related mortalities were significantly reduced. These results underscore the importance of continued liver disease surveillance post-SVR, while CVD management should parallel that of the general population.

**Supplementary Information:**

The online version contains supplementary material available at 10.1038/s41598-025-29972-6.

## Introduction

Infection with hepatitis C virus (HCV) causes chronic hepatitis and may progress to cirrhosis and hepatocellular carcinoma (HCC). HCV infection is a major public health concern and a leading cause of chronic liver disease, resulting in approximately 399,000 deaths annually^1^. The World Health Organization estimates that 58 million individuals are chronically infected and that 1.5 million new HCV infections occur annually (updated recommendations on treatment of adolescents and children with chronic HCV infection, and HCV simplified service delivery and diagnostics)^1^. In recent years, direct-acting antivirals (DAAs) have achieved sustained virologic response (SVR) rates exceeding 95%^2,3^, although treatment failure still occurs in some patients^4,5^. Several studies have demonstrated that patients who achieve SVR experience a significantly reduced incidence of new HCC compared with those who do not^6^. However, males and patients with advanced fibrosis remain at higher risk of developing HCC^7–9^.Conversely, DAA therapy has shown high efficacy even among older adults and patients with liver cirrhosis (LC)^10^. Viral eradication through DAA treatment has also been associated with reduced all-cause mortality^11–13^. Despite these advances, limited evidence exists regarding long-term prognosis and cause-specific mortality following viral eradication, particularly in comparison with the general population. Therefore, this study aimed to clarify life expectancy and specific causes of death after achieving SVR with DAAs.

## Results

### Patients characteristics

Baseline characteristics before DAA administration and at the end of treatment (EOT) are summarized in Table 1. The mean age was 67.0 years; 712 patients (40.6%) were male, 370 patients (21.1%) had LC, and 294 patients (16.8%) had diabetes mellitus (DM). The mean follow-up period was 59.2 months. Sofosbuvir (SOF)/velpatasvir (VEL) was administered to 19 patients with decompensated cirrhosis. Sixty-nine patients achieved SVR after retreatment: 20 patients with SOF/ledipasvir (LDV), 44 with glecaprevir (GLE)/pibrentasvir (PIB), and five with SOF/VEL + ribavirin (RBV). No patient had a history of organ transplantation.

**Table 1:** Baseline characteristics.

Characteristics	Patients (N = 1753)
Age, years	68 (61–75)
Male, n (%)	712 (40.6)
BMI, kg/m^2^ (n = 1409)	22.6 (20.6–24.9)
Liver cirrhosis, n (%)	370 (21.1)
Prior DAA therapy, none/ experience	1684 (96.1)/ 69 (3.9)
Genotype 1/ 2/ 1 + 2/ 3, n (%)	1350 (77.0)/ 401 (22.9)/ 1 (0.0)/ 1 (0.0)
Diabetes mellitus, n (%)	294 (16.8)
・DCV + ASV/・SOF/LDV/・OBV/PTV/r/・SOF + RBV ・GZR + EBR/・DCV/ASV/BCV/・GLE/PIB/・SOF/VEL, n (%)	361 (20.6)/ 506 (28.9)/ 110 (6.3)/ 200 (11.4) 110 (6.3)/ 12 (0.7)/ 422 (24.1)/ 32 (1.8)
HCV-RNA, logIU/mL	6.2 (5.6–6.6)
Observation period, months	60.9 (31.4–88.3)
Platelet counts, × 104/μL	15.8 (11.9–20.0)
Total bilirubin, mg/dL (n = 1750)	0.8 (0.6–1.0)
AST, U/L	39 (28–57)
ALT, U/L	36 (24–58)
GGT, U/L (n = 1753)	30 (19–51)
Creatinine, mg/dL (n = 1727)	0.70 (0.60–0.85)
Albumin, g/dL (n = 1712)	4.1 (3.8–4.3)
FIB-4 index	2.85 (1.98–4.63)
Hyaluronic acid, ng/mL (n = 1614)	82.5 (42.0–180.0)
AFP (before), ng/mL (n = 1731)	4.2 (2.7–7.7)
ALT (EOT), non WNL, n (%)	175 (10.0)
Albumin (EOT), g/dL (n = 1659)	4.1 (3.9–4.3)
AFP (EOT), ng/mL (n = 1612)	3.1 (2.1–5.1)

Continuous variables are presented as median (interquartile range, IQR).

Categorical variables are presented as number (percentage).

BMI, body mass index; DAA, direct-acting antivirals; DCV, daclatasvir; ASV, asunaprevir; SOF, sofosbuvir; LDV, ledipasvir; OBV, ombitasvir; PTV, paritaprevir; r, ritonavir; RBV, ribavirin; GZR, grazoprevir; EBR, elbasvir; BCV, beclabuvir; GLE, glecaprevir; PIB, pibrentasvir; VEL, velpatasvir, AST, aspartate aminotransferase; ALT, alanine aminotransferase; GGT, γ-glutamyltransferase; AFP, alpha-fetoprotein; EOT, end of treatment; WNL, within normal limit; FIB-4, Fibrosis-4.

### Development of HCC and cause of death

During the course of the study, 108 patients developed HCC, and 122 patients died. A detailed breakdown of causes of death is shown in Table 2. Among 34 liver disease-related (LDR) deaths, 21 were due to HCC, four to intrahepatic cholangiocarcinoma, four to liver failure, two to variceal bleeding, one to cystadenocarcinoma, and one to an unknown cause. Other causes of death included cardiovascular disease (CVD) in 23 patients (13 cardiovascular, 10 cerebrovascular), gastrointestinal disease in 13, and respiratory disease in nine. Malignancy accounted for 48 deaths, of which 22 were due to extrahepatic cancers. Two accidental deaths occurred: one suicide and one work-related accident.

**Table 2:** Causes of death.

Organs	Diseases	Number of deaths
Liver (n = 34)	Hepatocellular carcinoma	21
	Intrahepatic cholangiocarcinoma	4
	Liver failure	4
	Varix	2
	Cystadenocarcinoma	1
	Unknown	1
Cardiovascular (n = 23)	Cerebral hemorrhage	8
	Heart failure	7
	Cardiac infarction	4
	Cerebral infarction	2
	Pulmonary hypertension	1
	Pulmonary embolism	1
Digestive (n = 13)	Pancreatic carcinoma (IPMN)	5 (1)
	Intra-abdominal hemorrhage	3
	Colo-rectal cancer	2
	Perforated digestive tract	1
	Cholangiocarcinoma	1
	Gastric cancer	1
Respiratory (n = 9)	Infectious pneumonia	3
	Lung cancer	3
	Interstitial pneumonia	2
	Respiratory failure	1
Oral cavity (n = 2)	Carcinoma of tongue	1
	Gingival carcinoma	1
Blood (n = 2)	Myelodysplastic syndrome	1
	Leukemia	1
Renal, urinary tract (n = 3)	Renal failure	2
	Bladder cancer	1
Infection (n = 2)	Sepsis	1
	Infection	1
Mammary gland (n = 2)	Breast cancer	1
	Extramammary Paget’s disease	1
Uterus (n = 1)	Cancer of the uterine body	1
Primary undetermined (n = 6)	Cancer of unknown primary origin	2
	Multiple organ failure	1
	Heatstroke	1
	Accident	2
Unknown (n = 25)		25

IPMN, intraductal papillary mucinous neoplasm.

### Mortality rate and factors associated with mortality

**Fig. 1:**
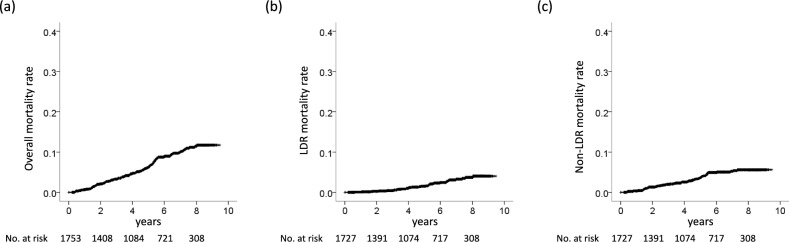
Mortality rate. (**a**) overall mortality rate, (**b**) LDR mortality rate, (**c**) non-LDR mortality rate. LDR, liver-disease related.

Overall mortality rates were 3.3%, 6.2%, and 10.1% at 3, 5, and 7 years, respectively (Fig. 1a). LDR and non-LDR mortality rates were 0.4% and 2.0% at 3 years, 1.5% and 3.7% at 5 years, and 3.3% and 5.3% at 7 years, respectively (Fig. 1b,c). A full multivariable model including all covariates identified age, male sex, and creatinine level as factors associated with overall mortality (Table 3). A reduced model using the Akaike Information Criterion (AIC) identified age, male sex, cirrhosis, creatinine level, and albumin level before therapy as factors associated with overall mortality (Table 3). LDR mortality was associated with age, male sex, total bilirubin level, and alpha-fetoprotein (AFP) level at EOT in the full multivariable model including all covariates (Table 4). A reduced model using the AIC identified age, male sex, total bilirubin level, Fibrosis-4 (FIB-4) index, albumin level at EOT, and AFP level at EOT as factors associated with LDR mortality (Table 4). Non-LDR mortality was associated with age and male sex in the full multivariable model including all covariates (Table 5). A reduced model using the AIC identified age, male sex, and albumin level before therapy as factors associated with non-LDR mortality (Table 5).

**Table 3:** Factors associated with cause of death.

All (n = 1419)	Univariable			Multivariable (AIC = 1273)			Multivariable (AIC = 1262)		
	Hazard ratio	95%CI	*p* value	Hazard ratio	95% CI	*p* value	Hazard ratio	95% CI	*p* value
Age	1.087	1.064–1.111	< 0.001	1.081	1.053–1.110	< 0.001	1.087	1.060–1.114	< 0.001
Male sex	1.924	1.348–2.748	< 0.001	2.275	1.487–3.481	< 0.001	2.297	1.533–3.441	< 0.001
Presence of liver cirrhosis	2.987	2.086–4.278	< 0.001	1.702	0.968–2.993	0.065	2.013	1.261–3.212	0.003
Presence of diabetes mellitus	1.838	1.228–2.752	0.003	1.325	0.827–2.124	0.242			
History of DAA therapy	1.167	0.477–2.860	0.735	0.737	0.178–3.053	0.673			
Platelet count	0.922	0.892–0.953	< 0.001	0.991	0.934–1.052	0.773			
Total bilirubin	1.626	1.294–2.044	< 0.001	1.253	0.838–1.874	0.271			
ALT	0.995	0.989–1.001	0.078	0.992	0.984–1.001	0.084			
GGT	0.999	0.996–1.003	0.690	1.001	0.997–1.006	0.517			
Creatinine	1.123	1.028–1.226	0.010	1.144	1.013–1.291	0.030	1.147	1.029–1.279	0.013
Albumin	0.249	0.176–0.353	< 0.001	0.562	0.281–1.122	0.102	0.422	0.250–0.711	< 0.001
AFP	1.000	0.995–1.005	0.923	0.974	0.949–1.000	0.054	0.977	0.952–1.002	0.070
Hyaluronic acid	1.001	1.000–1.001	< 0.001	1.000	1.000–1.001	0.885			
FIB-4 index	1.127	1.091–1.164	< 0.001	1.047	0.948–1.157	0.365			
EOT-Albumin	0.220	0.148–0.328	< 0.001	0.820	0.422–1.592	0.557			
EOT-AFP	1.015	1.004–1.026	0.005	1.013	0.999–1.026	0.060			
EOT-ALT (WNL)	1.020	0.594–1.752	0.944	0.937	0.500–1.755	0.839			

CI, confidence interval; EOT, end of treatment; AIC, Akaike Information Criterion; DAAs, direct-acting antivirals; ALT, alanine aminotransferase; GGT, γ-glutamyltransferase; AFP, alpha-fetoprotein; EOT, end of treatment; WNL, within normal limit; FIB-4, Fibrosis-4.

**Table 4:** Factors associated with liver disease-related deaths.

Liver disease-related death (n = 1298)	Univariable			Multivariable (AIC = 284)			Multivariable (AIC = 272)		
	Hazard ratio	95% CI	*p* value	Hazard ratio	95% CI	*p* value	Hazard ratio	95% CI	*p* value
Age	1.077	1.035–1.121	< 0.001	1.082	1.025–1.142	0.004	1.071	1.018–1.126	0.008
Male sex	1.643	0.839–3.218	0.148	3.001	1.222–7.370	0.017	2.763	1.197–6.380	0.017
Presence of liver cirrhosis	5.239	2.661–10.313	< 0.001	2.538	0.720–8.939	0.147			
Presence of diabetes mellitus	1.125	0.466–2.717	0.793	0.727	0.226–2.337	0.592			
History of DAA therapy	1.826	0.436–7.643	0.410	2.483	0.303–20.312	0.397			
Platelet count	0.867	0.810–0.929	< 0.001	1.012	0.892–1.149	0.850			
Total bilirubin	2.397	1.827–3.145	< 0.001	2.405	1.448–3.994	0.001	2.264	1.374–3.733	0.001
ALT	0.988	0.974–1.002	0.098	0.979	0.957–1.002	0.071	0.981	0.961–1.002	0.079
GGT	1.002	0.997–1.006	0.540	1.004	0.998–1.011	0.192			
Creatinine	1.024	0.802–1.308	0.848	1.120	0.783–1.602	0.534			
Albumin	0.125	0.068–0.229	< 0.001	0.862	0.196–3.788	0.845			
AFP	1.001	0.993–1.009	0.865	0.984	0.957–1.013	0.282			
Hyaluronic acid	1.001	1.000–1.001	0.003	0.999	0.997–1.001	0.188	0.999	0.997–1.000	0.101
FIB-4 index	1.185	1.130–1.243	< 0.001	1.133	0.992–1.294	0.066	1.137	1.035–1.249	0.008
EOT-albumin	0.081	0.041–0.161	< 0.001	0.231	0.050–1.070	0.061	0.147	0.053–0.404	< 0.001
EOT-AFP	1.015	1.004–1.026	0.005	1.020	1.007–1.034	0.003	1.020	1.007–1.033	0.003
EOT-ALT (WNL)	1.475	0.610–3.567	0.389	1.619	0.546–4.804	0.385			

CI, confidence interval; EOT, end of treatment; AIC, Akaike Information Criterion; DAAs, direct-acting antivirals; ALT, alanine aminotransferase; GGT, γ-glutamyltransferase; AFP, alpha-fetoprotein; EOT, end of treatment; WNL, within normal limit; FIB-4, Fibrosis-4.

**Table 5:** Factors associated with non-liver disease-related death.

Non-liver disease-related death (n = 1398)	Univariable			Multivariable (AIC = 717)			Multivariable (AIC = 705)		
	Hazard ratio	95% CI	*p* value	Hazard ratio	95% CI	*p* value	Hazard ratio	95% CI	*p* value
Age	1.087	1.055–1.120	< 0.001	1.082	1.044–1.122	< 0.001	1.081	1.045–1.117	< 0.001
Male sex	2.261	1.365–3.744	0.002	2.256	1.261–4.035	0.006	2.686	1.543–4.677	< 0.001
Presence of liver cirrhosis	2.374	1.418–3.974	0.001	1.497	0.702–3.190	0.296			
Presence of diabetes mellitus	2.325	1.355–3.989	0.002	1.597	0.866–2.948	0.134			
History of DAA therapy	0.865	0.211–3.542	0.841	< 0.001	0–8.87E188	0.960			
Platelet count	0.938	0.896–0.982	0.006	0.959	0.880–1.045	0.335	0.950	0.896–1.007	0.082
Total bilirubin	0.750	0.374–1.506	0.419	0.543	0.228–1.293	0.168	0.537	0.225–1.282	0.162
ALT	0.999	0.993–1.005	0.697	1.000	0.991–1.010	0.920			
GGT	0.998	0.993–1.004	0.568	1.002	0.996–1.008	0.600			
Creatinine	1.162	1.044–1.293	0.006	1.090	0.933–1.274	0.279	1.120	0.975–1.286	0.109
Albumin	0.300	0.182–0.494	< 0.001	0.433	0.171–1.100	0.079	0.368	0.185–0.733	0.004
AFP	0.979	0.992–1.008	0.979	0.965	0.901–1.034	0.313	0.961	0.914–1.010	0.117
Hyaluronic acid	1.001	1.000–1.001	0.001	1.000	1.000–1.001	0.265			
FIB-4 index	1.099	1.043–1.159	< 0.001	0.975	0.823–1.155	0.771			
EOT-Albumin	0.302	0.170–0.537	< 0.001	1.089	0.451–2.626	0.850			
EOT-AFP	0.935	0.848–1.031	0.177	0.963	0.830–1.117	0.619			
EOT-ALT (WNL)	0.954	0.434–2.096	0.907	0.904	0.380–2.148	0.819			

CI, confidence interval; EOT, end of treatment; AIC, Akaike Information Criterion; DAAs, direct-acting antivirals; ALT, alanine aminotransferase; GGT, γ-glutamyltransferase; AFP, alpha-fetoprotein; EOT, end of treatment; WNL, within normal limit; FIB-4, Fibrosis-4.

### Standardized mortality ratios (SMRs) for all-cause and each cause of death

SMRs for this cohort are shown in Table 6. The SMR for all-cause mortality was 1.050 (95% confidence interval [CI] 0.872–1.254), not significantly different from that of the general population (*p* = 0.588). Stratification by age, sex, and presence of cirrhosis demonstrated no significant differences for age or sex (Supplementary Table 1). However, the SMR was significantly lower in patients without LC at 0.762 (95% CI 0.594–0.963, *p* = 0.023) and significantly higher in patients with LC at 2.139 (95% CI 1.598–2.805, *p* < 0.001) and in those with DM at 1.451 (95% CI 0.993–2.049, *p* = 0.034) (Supplementary Table 1).

**Table 6:** Standardized mortality ratio for all-cause mortality and each cause of death.

	Sum of observation periods (person years)	Observed deaths (n)	Expected deaths (n)	SMR	95% CI	*p* value
All-cause mortality	8683.3	122	116.2	1.050	0.872–1.254	0.588
LDR death	8592.7	34	4.3	7.819	5.415–10.926	< 0.001
Non-LDR death	8592.7	62	109.6	0.566	0.434–0.725	< 0.001
Malignancy death	8592.7	48	39.4	1.219	0.899–1.616	0.169
Hepatic malignancy death	8592.7	26	2.2	11.612	7.586–17.015	< 0.001
Extrahepatic malignancy death	8592.7	22	37.1	0.593	0.371–0.897	0.013
Cardiovascular death	8592.7	23	26.0	0.884	0.560–1.326	0.554

CI, confidence interval; LDR, liver disease-related; SMR, standardized mortality ratio.

Further comparisons of SMRs stratified by age and sex revealed that in patients without LC, the SMR was significantly lower among those without DM at 0.692 (95% CI 0.517–0.908, *p* = 0.008) (Supplementary Table 1). No significant differences in SMR were observed by age, sex, presence of DM, or chronic kidney disease (CKD) stage. In contrast, in patients with LC, SMRs were significantly elevated regardless of age, sex, DM status, or CKD stage (Supplementary Table 1).

### SMR for cause-specific mortality

Cause-specific SMRs were calculated among individuals with confirmed causes of death (Table 6). The SMR for LDR-related mortality was markedly elevated at 7.819 (95% CI 5.415–10.926, *p* < 0.001). Stratified analyses by age, sex, LC, DM, and CKD stage demonstrated consistently high SMRs across all subgroups (Supplementary Table 2). In contrast, the SMR for non-LDR deaths was significantly lower at 0.566 (95% CI 0.434–0.725, *p* < 0.001) (Supplementary Table 2). Stratified analyses by age, sex, and non-cirrhotic status also showed significantly lower SMRs, whereas among individuals with cirrhosis, DM, or CKD stage ≥ 3, no significant differences were observed (Supplementary Table 2).

The SMR for malignancy-related deaths was 1.219, revealing no significant difference overall (95% CI 0.899–1.616, *p* = 0.169) (Table 6). Stratified analyses based on presence of cirrhosis and DM yielded significantly higher SMRs at 2.476 (95% CI 1.512–3.823, *p* < 0.001) and 1.733 (95% CI 0.923–2.984, *p* = 0.045), respectively (Supplementary Table 2). However, stratified analyses based on age, sex, absence of cirrhosis, absence of DM, and CKD stage status revealed no significant differences (Supplementary Table 2).

The SMR for deaths due to hepatic malignancies was strikingly elevated at 11.612 (95% CI 7.586–17.015, *p* < 0.001) (Table 6), remaining significantly higher across all strata of age, sex, cirrhosis, DM, and CKD status (Supplementary Table 2). Conversely, the SMR for deaths due to extrahepatic malignancies was significantly reduced at 0.593 (95% CI 0.371–0.897, *p* = 0.013) (Table 6). This reduction was particularly evident among individuals aged ≥ 75 years (0.529, 95% CI 0.254–0.972, *p* = 0.040), males (0.520, 95% CI 0.250–0.957, *p* = 0.036), those without cirrhosis (0.508, 95% CI 0.285–0.839, *p* = 0.008), those without DM (0.465, 95% CI 0.254–0.781, *p* = 0.003), and those with CKD stage < 3 (0.536, 95% CI 0.286–0.917, *p* = 0.022) (Supplementary Table 2).

The SMR for CVD mortality was 0.884 (95% CI 0.560–1.326, *p* = 0.554) (Table 6). Stratified analyses by age, sex, cirrhosis, DM, and CKD stage revealed no significant differences (Supplementary Table 2).

## Discussion

Regarding prognosis after achieving SVR for HCV, several studies have reported reductions in all-cause, LDR, and non-LDR mortalities compared with that in patients without SVR^14,15^. In the present cohort, however, liver disease remained the leading cause of death, accounting for 34 cases (31.5%), with 21 cases due to HCC. Multivariable analysis demonstrated that LDR mortality was associated with age, male sex, albumin, bilirubin, and EOT-AFP, consistent with previously reported HCC risk factors^7,16,17^. Elevated SMRs for LDR deaths were observed even among patients without LC, DM, or CKD (Table 6). These findings underscore the importance of ongoing surveillance for liver disease, as recommended by current clinical guidelines^18^.

Most deaths in this cohort were due to non-LDR causes. Multivariable analysis identified creatinine as a predictor of all-cause mortality, suggesting an association with lifestyle-related conditions. Few studies have evaluated cause-specific mortality due to extrahepatic malignancies or CVD in patients with SVR compared with that in the general population. Notably, the SMR for non-LDR deaths, particularly extrahepatic malignancies, was significantly lower. This reduction may reflect early detection through routine medical follow-up. In contrast, the SMR for CVD mortality did not differ from that of the general population, regardless of age, sex, cirrhosis, or DM status, suggesting that CVD should be monitored in the same manner as in the general population.

Bruno et al. showed that the survival rate of patients with HCV cirrhosis and SVR is similar to that of the general population^19^. Similarly in Japan, Miuma et al. reported that in patients with DAA-induced SVR, the SMR was 0.96 for those without advanced fibrosis or cirrhosis and 0.92 for those with advanced fibrosis or LC^20^. Conversely, Innes et al. showed that all-cause mortality was 1.9 times more frequent for SVR patients than for the general population^21^.They noted that most of the surplus mortality was due to drug-related causes and liver cancer. The differences compared with the present study may reflect higher mortality, differing patient characteristics, and a longer observation period. In our cohort, LDR deaths were more frequent among individuals with cirrhosis, whereas deaths unrelated to liver disease occurred at similar rates.

Obi et al. analyzed 651 patients who achieved SVR across Japan (median follow-up, 5.44 years) and reported survival rates of 99.3% at 1 year, 96.5% at 3 years, and 94.4% at 5 years, with age- and sex-adjusted SMRs comparable to those of the general Japanese population^22^.They also noted that malignancies of other organs occurred as frequently as that of HCC^22^, although comparisons with the general population were not reported.

Calvaruso et al. assessed cardiovascular outcomes after SVR in 4,307 patients and found improved hepatic and cardiovascular survival among those treated with DAAs^23^. In that study, SVR, CKD stage ≥ 3, and DM were significantly associated with CVD mortality^23^. Several prior investigations and meta-analyses suggest that HCV infection increases CVD risk, particularly among individuals with pre-existing conditions such as DM and hypertension^24^. In contrast, the present study compared SVR patients with the general population and found no excess CVD mortality, even among those with cirrhosis or CKD. These findings indicate that achieving SVR reduces CVD risk to levels similar to those in the general population, supporting the need for routine CVD follow-up consistent with standard practice.

This study has some limitations. First, the cause of death was unknown for some non-LDR patients. Second, the number of patients and the follow-up period remain insufficient to fully evaluate long-term mortality. Larger cohorts with extended follow-up are necessary for more definitive analysis. Third, data on lifestyle-related diseases other than DM, such as hypertension and dyslipidemia, and alcohol intake were not collected. Nonetheless, because CVD SMRs were comparable with those of the general population, careful monitoring of CVD remains warranted after HCV-SVR.

In conclusion, compared with the general population, all-cause mortality in patients with HCV-SVR was similar, whereas LDR mortality was significantly elevated. Conversely, non-LDR mortality and deaths due to extrahepatic malignancies were reduced, while CVD mortality was comparable. These results highlight the continued importance of liver disease management after SVR, along with standard CVD monitoring.

## Material and methods

### Data collection

**Fig. 2:**
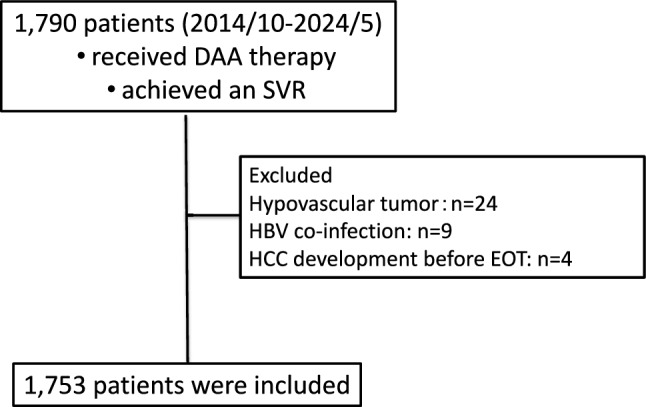
Study flow.

This observational study was conducted at 21 facilities of the Kagoshima Liver Study Group in Japan. The enrollment of the study population is presented in Fig. 2. Of 1790 patients without a history of HCC who achieved SVR following DAA therapy between October 2014 and May 2024, 1753 patients were included. Patients with hepatitis B surface antigen positivity or who developed cancer before the completion of DAA therapy were excluded. Patients with hypovascular tumors in the liver were also excluded because such lesions frequently progress to HCC despite HCV eradication with DAA therapy^25,26^. Data regarding survival status, cause of death, and mortality were collected from medical records. The study protocol adhered to the ethical principles of the Declaration of Helsinki and was approved by the Kagoshima University Hospital Clinical Research Ethics Committee and the ethics committees of all participating institutions (approval numbers: 150138, 170,199, 190,297). Written informed consent was obtained from all enrolled patients.

HCV RNA levels were measured via TaqMan polymerase chain reaction (PCR), which has a lower quantitation limit of 1.2 log IU/mL. LC was diagnosed by liver specialists at each institution based on a composite assessment of the following: pathological assessment (METAVIR F4), liver stiffness measurement > 12.5 kPa^27^, platelet count < 100 × 10^3^ μL, evaluation of symptoms of liver failure (varices, ascites, or encephalopathy), or morphological assessment (blunted nodular edge and splenomegaly) in imaging modalities such as ultrasound, Computed Tomography, or Magnetic Resonance Imaging. DM was defined as treatment with antidiabetic medications, fasting blood glucose level ≥ 126 mg/dL, 2-h plasma glucose level ≥ 200 mg/dL during a 75-g oral glucose tolerance test, and/or hemoglobin A1c concentration ≥ 6.5% on a single test or on two or more separate occasions^28^. No patients with prediabetes or normoglycemia received antidiabetic medications for other indications. Non-LDR deaths were defined as deaths from causes other than liver disease when the cause was confirmed; accidental deaths were included as non-LDR. CVD deaths were classified according to Japanese guidelines and included coronary heart disease^29^, aortic disease^30^, and cerebrovascular disease^31^. CKD stage was determined based on the estimated glomerular filtration rate (eGFR)^32^. eGFR was calculated using the Modification of Diet in Renal Disease (MDRD) Study-based Japanese correction formula^33^, which is the “Japanese coefficient” version of the MDRD Study equation validated in Japanese populations.

### Treatment protocol

The treatment regimens included daclatasvir (DCV) plus asunaprevir (ASV) for 24 weeks; SOF plus LDV for 12 weeks; ombitasvir (OBV), paritaprevir (PTV), and ritonavir (r) for 12 weeks; SOF plus RBV for 12 weeks; elbasvir (EBR) plus grazoprevir (GZR) for 12 weeks; DCV, ASV, and beclabuvir (BCV) for 12 weeks; glecaprevir (GLE) plus pibrentasvir (PIB) for 8 or 12 weeks; and SOF plus velpatasvir (VEL) for 12 weeks. All treatments were administered according to the Japanese guidelines for chronic HCV infection^34^. The observation period commenced at the EOT.

### Statistical analyses

Analyses were conducted using IBM SPSS Statistics software (version 22; IBM, Armonk, NY, USA). Categorical variables were compared using the chi-squared test or Fisher’s exact test, as appropriate. Continuous variables were analyzed with the Mann–Whitney U test or the Kruskal–Wallis test. Mortality was assessed using the Kaplan–Meier method and compared using the log-rank test. All statistical tests were two-tailed, and a *p*-value < 0.05 was considered statistically significant. Factors associated with mortality were examined using a Cox proportional hazards model. We first constructed a full multivariable Cox model including all covariates. Then, we derived a reduced model by stepwise selection based on the AIC. The final model with the lowest AIC was considered optimal. Covariates included age, sex, presence of LC, presence of DM, history of DAA therapy, pre-treatment laboratory values (platelet count, total bilirubin, alanine aminotransferase [ALT], γ-glutamyl transpeptidase [GGT], creatinine, albumin, AFP, hyaluronic acid, and FIB-4 index), and albumin, AFP, and ALT levels at EOT. Mortality rates adjusted for sex and age, were compared with those of the general Japanese population using SMRs. The SMR was calculated as the ratio of observed to expected deaths, where expected deaths were obtained by multiplying person-years at risk in each stratum by the corresponding mortality rate of the reference population and summing across all strata. Population data on age group, sex, mortality, and cause of death were obtained from the 2023 Population Survey Report of the Ministry of Health, Labour, and Welfare (eSTAT; Supplementary Table)^35^. The 95% CIs were calculated based on observed and expected deaths.

## Supplementary Information

Supplementary Information 1.

Supplementary Information 2.

## Data Availability

The data supporting the findings of this study are available from the corresponding author upon reasonable request.

## Abbreviations

HCV: Hepatitis C virus
DAAs: Direct-acting antivirals
SVR: Sustained virologic response
HCC: Hepatocellular carcinoma
LC: Liver cirrhosis
DM: Diabetes mellitus
LDR: Liver disease-related
CVD: Cardiovascular disease
eGFR: Estimated glomerular filtration rate
EOT: End of treatment
AFP: Alpha-fetoprotein
SMR: Standardized mortality ratio
CI: Confidence interval
AIC: Akaike information criterion
