# Supplementary material for: Cause-specific mortality following sustained virologic response in hepatitis C patients treated with direct-acting antivirals: a standardized mortality ratio analysis
Source: Sci Rep. 2025 Nov 27;15:45560. doi: 10.1038/s41598-025-29972-6 (PMC12749750; doi:10.1038/s41598-025-29972-6)
Supplement: Supplementary file 1 — Supplementary Information 1. [file 41598_2025_29972_MOESM1_ESM.docx]

Supplementary Table1. Standardized mortality ratio for all-cause of death stratified by cirrhosis

|  | | The sum of observation periods (person years) | Observed deaths  (n) | Expected deaths (n) | SMR | 95%CI | P value |
| --- | --- | --- | --- | --- | --- | --- | --- |
| All-cause of death in patients without LC | | 6932.4 | 70 | 91.9 | 0.762 | 0.594-0.963 | 0.023 |
| Age | <75 years | 5,277.0 | 29 | 37.2 | 0.779 | 0.521–1.118 | 0.176 |
|  | >75 years | 1,655.5 | 41 | 54.6 | 0.751 | 0.539–1.019 | 0.066 |
| Sex | Male | 2,749.0 | 41 | 50.9 | 0.805 | 0.578–1.092 | 0.165 |
|  | Female | 4,183.5 | 29 | 40.9 | 0.708 | 0.474–1.017 | 0.062 |
| DM | Absence | 5,829.0 | 52 | 75.1 | 0.692 | 0.517–0.908 | 0.008 |
|  | Presence | 1,103.5 | 18 | 16.7 | 1.075 | 0.637–1.698 | 0.760 |
| CKD | ≥Stage 3 | 1,530.1 | 25 | 33.4 | 0.749 | 0.485–1.105 | 0.147 |
|  | <Stage 3 | 5,272.1 | 44 | 56.9 | 0.774 | 0.562–1.039 | 0.088 |
| All-cause of death in patients with LC | | 1750.9 | 52 | 24.3 | 2.139 | 1.598-2.805 | <0.001 |
| Age | <75 years | 1,234.0 | 26 | 8.6 | 3.023 | 1.975–4.429 | <0.001 |
|  | >75 years | 516.9 | 26 | 15.7 | 1.655 | 1.081–2.425 | 0.009 |
| Sex | Male | 579.1 | 25 | 11.6 | 2.162 | 1.399–3.191 | <0.001 |
|  | Female | 1,171.8 | 27 | 12.7 | 2.118 | 1.396–3.082 | <0.001 |
| DM | Absence | 1,445.2 | 38 | 19.0 | 1.999 | 1.415–2.744 | <0.001 |
|  | presence | 305.7 | 14 | 5.3 | 2.642 | 1.444–4.432 | <0.001 |
| CKD | ≥Stage 3 | 441.1 | 26 | 8.8 | 2.942 | 1.922–4.311 | <0.001 |
|  | <Stage 3 | 1,271.7 | 25 | 15.3 | 1.639 | 1.060–2.419 | 0.013 |

LDR, liver disease-related; SMR, standardized mortality ratio; CI, confidence interval.

LC, liver cirrhosis; DM, diabetes mellitus; CKD, chronic kidney disease

Supplementary Table 2. Standardized mortality ratio for each cause of death

|  | | The sum of observation periods (person years) | Observed deaths (n) | Expected deaths (n) | SMR | 95%CI | P value |
| --- | --- | --- | --- | --- | --- | --- | --- |
| LDR death | |  |  |  |  |  |  |
| Age | <75 years | 6,487.9 | 16 | 2.3 | 6.866 | 3.924–11.149 | <0.001 |
|  | >75 years | 2,104.8 | 18 | 2.0 | 8.920 | 5.287–14.098 | <0.001 |
| Sex | Male | 3,285.0 | 17 | 2.7 | 6.270 | 3.653–10.039 | <0.001 |
|  | Female | 5,307.6 | 17 | 1.6 | 10.384 | 6.049–16.625 | <0.001 |
| Cirrhosis | Non-LC | 6,873.6 | 15 | 3.5 | 4.314 | 2.415–7.116 | <0.001 |
|  | LC | 1,719.0 | 19 | 0.9 | 21.797 | 13.123–34.039 | <0.001 |
| DM | Absence | 7,214.9 | 28 | 3.5 | 8.039 | 5.342–11.618 | <0.001 |
|  | presence | 1,377.7 | 6 | 0.9 | 6.935 | 2.545–15.095 | <0.001 |
| CKD | ≥Stage 3 | 1,938.1 | 13 | 1.4 | 9.335 | 4.970–15.963 | <0.001 |
|  | <Stage 3 | 6,486.2 | 19 | 2.9 | 6.613 | 3.982–10.327 | <0.001 |
| Non-LDR death | |  |  |  |  |  |  |
| Age | <75 years | 6,487.9 | 30 | 43.2 | 0.694 | 0.468–0.991 | 0.044 |
|  | >75 years | 2,104.8 | 32 | 66.4 | 0.482 | 0.330–0.680 | <0.001 |
| Sex | Male | 3,285.0 | 36 | 58.4 | 0.616 | 0.432–0.853 | 0.003 |
|  | Female | 5,307.6 | 26 | 51.2 | 0.507 | 0.331–0.744 | <0.001 |
| Cirrhosis | Non-LC | 6,873.6 | 39 | 87.0 | 0.448 | 0.319–0.613 | <0.001 |
|  | LC | 1,719.0 | 23 | 22.6 | 1.016 | 0.644–1.524 | 0.941 |
| DM | Absence | 7,214.9 | 43 | 88.9 | 0.484 | 0.350–0.651 | <0.001 |
|  | presence | 1,377.7 | 19 | 20.7 | 0.628 | 0.334-1.074 | 0.090 |
| CKD | ≥Stage 3 | 1,938.1 | 28 | 40.3 | 0.695 | 0.462-1.005 | 0.053 |
|  | <Stage 3 | 6,486.2 | 34 | 67.6 | 0.503 | 0.348-0.703 | <0.001 |
| Malignancy death | |  |  |  |  |  |  |
| Age | <75 years | 6,487.9 | 23 | 19.2 | 1.196 | 0.758–1.794 | 0.391 |
|  | >75 years | 2,104.8 | 25 | 20.1 | 1.242 | 0.804–1.833 | 0.278 |
| Sex | Male | 3,285.0 | 23 | 20.6 | 1.114 | 0.706–1.672 | 0.604 |
|  | Female | 5,307.6 | 25 | 18.7 | 1.335 | 0.864–1.971 | 0.147 |
| Cirrhosis | Non-LC | 6,873.6 | 28 | 31.3 | 0.895 | 0.595–1.293 | 0.556 |
|  | LC | 1,719.0 | 20 | 8.1 | 2.476 | 1.512–3.823 | <0.001 |
| DM | Absence | 7,214.9 | 35 | 31.9 | 1.098 | 0.765–1.527 | 0.579 |
|  | presence | 1,377.7 | 13 | 7.5 | 1.733 | 0.923–2.984 | 0.045 |
| CKD | ≥Stage 3 | 1,938.1 | 19 | 13.0 | 1.462 | 0.880–2.284 | 0.096 |
|  | <Stage 3 | 6,486.2 | 27 | 25.7 | 1.052 | 0.694–1.531 | 0.791 |
| Hepatic malignancy death | |  |  |  |  |  |  |
| Age | <75 years | 6,487.9 | 11 | 1.0 | 10.764 | 5.374–19.260 | <0.001 |
|  | >75 years | 2,104.8 | 15 | 1.2 | 12.324 | 6.898–20.327 | <0.001 |
| Sex | Male | 3,285.0 | 13 | 1.4 | 9.083 | 4.836–15.532 | <0.001 |
|  | Female | 5,307.6 | 13 | 0.8 | 16.094 | 8.570–27.522 | <0.001 |
| Cirrhosis | Non-LC | 6,873.6 | 13 | 1.8 | 7.268 | 3.870–12.428 | <0.001 |
|  | LC | 1,719.0 | 13 | 0.5 | 28.869 | 15.372–49.367 | <0.001 |
| DM | Absence | 7,214.9 | 21 | 1.8 | 11.776 | 7.290–18.001 | <0.001 |
|  | presence | 1,377.7 | 5 | 0.5 | 10.971 | 3.562–25.602 | <0.001 |
| CKD | ≥Stage 3 | 1,938.1 | 10 | 0.8 | 12.840 | 6.157-23.613 | <0.001 |
|  | <Stage 3 | 6,486.2 | 14 | 1.4 | 9.869 | 5.396-16.559 | <0.001 |
| Extrahepatic malignancy death | |  |  |  |  |  |  |
| Age | <75 years | 6,487.9 | 12 | 18.2 | 0.659 | 0.340–1.151 | 0.145 |
|  | >75 years | 2,104.8 | 10 | 18.9 | 0.529 | 0.254–0.972 | 0.040 |
| Sex | Male | 3,285.0 | 10 | 19.2 | 0.520 | 0.250–0.957 | 0.036 |
|  | Female | 5,307.6 | 12 | 17.9 | 0.670 | 0.346–1.170 | 0.162 |
| Cirrhosis | Non-LC | 6,873.6 | 15 | 29.5 | 0.508 | 0.285–0.839 | 0.008 |
|  | LC | 1,719.0 | 7 | 7.6 | 0.918 | 0.369–1.891 | 0.820 |
| DM | Absence | 7,214.9 | 14 | 30.1 | 0.465 | 0.254–0.781 | 0.003 |
|  | presence | 1,377.7 | 8 | 7.0 | 1.136 | 0.490-2.238 | 0.719 |
| CKD | ≥Stage 3 | 1,938.1 | 9 | 12.2 | 0.737 | 0.337–1.399 | 0.358 |
|  | <Stage 3 | 6,486.2 | 13 | 24.2 | 0.536 | 0.286–0.917 | 0.022 |
| Cardiovascular death | |  |  |  |  |  |  |
| Age | <75 years | 6,487.9 | 13 | 9.9 | 1.310 | 0.698–2.240 | 0.329 |
|  | >75 years | 2,104.8 | 10 | 16.1 | 0.621 | 0.298–1.143 | 0.129 |
| Sex | Male | 3,285.0 | 16 | 14.0 | 1.141 | 0.652–1.853 | 0.597 |
|  | Female | 5,307.6 | 7 | 12.0 | 0.584 | 0.235–1.202 | 0.149 |
| Cirrhosis | Non-LC | 6,873.6 | 14 | 20.6 | 0.679 | 0.371–1.139 | 0.144 |
|  | LC | 1,719.0 | 9 | 5.4 | 1.671 | 0.764–3.171 | 0.120 |
| DM | Absence | 7,214.9 | 17 | 21.1 | 0.805 | 0.469–1.289 | 0.371 |
|  | presence | 1,377.7 | 6 | 4.9 | 1.223 | 0.449–2.662 | 0.622 |
| CKD | ≥Stage 3 | 1,938.1 | 10 | 9.7 | 1.030 | 0.494–1.895 | 0.924 |
|  | <Stage 3 | 6,486.2 | 13 | 15.9 | 0.817 | 0.435–1.397 | 0.465 |

LDR, liver disease-related; SMR, standardized mortality ratio; CI, confidence interval.

LC, liver cirrhosis; DM, diabetes mellitus; CKD, chronic kidney disease
